# Epidemiology of pediatric eye injuries requiring hospitalization in rural areas of Wenzhou and Changsha, China: a 10-year retrospective study

**DOI:** 10.1186/s12886-020-01363-7

**Published:** 2020-03-14

**Authors:** Chunyan Li, Yaoyao Lin, Haishao Xiao, Huan Lin, Yanyan Chen, Minhui Dai

**Affiliations:** 1 Ophthalmology Department, Xiangya Hospital, Central South University, Changsha, Hunan China; 2 Nursing Department of Central South University, Changsha, Hunan China; 3 School of Optometry and Ophthalmology, Wenzhou Medical University, Wenzhou, Zhejiang China; 4 The Eye Hospital, Wenzhou Medical University, Wenzhou, Zhejiang China

**Keywords:** Pediatric, Eye injury, Rural, Epidemiology, Visual acuity

## Abstract

**Background:**

The aim of this study was to review the demographic and characteristic distribution data of serious rural pediatric eye injuries in Wenzhou and Changsha, located in Zhejiang Province in East China and Hunan Province in Central China.

**Methods:**

This retrospective study included hospitalized pediatric patients aged < 18 years with eye injuries at the Eye Hospital of Wenzhou Medical University and Xiangya Hospital of Central South University from January 2008 to December 2017. Demographic data, injury types, injury causes, and initial and final visual acuity (VA) were recorded and analyzed. The ocular trauma score (OTS) was calculated to assess the severity of injury and evaluate the prognosis. All patient data were obtained from the medical record systems.

**Results:**

In total, 1125 children were hospitalized during the 10-year period; 830 (73.8%) were males and 295 (26.2%) were females. The majority of the patients were aged 3 to 8 years (57.4%, *n* = 646). Among mechanical injuries (*n* = 1007), penetrating injury was the most common (68.4%, *n* = 689), followed by contusion (17.2%, *n* = 173) and rupture (8.1%, *n* = 82). Overall, the top three injury causes were sharp objects (*n* = 544, 48.4%), blunt objects (*n* = 209, 18.6%) and fireworks (*n* = 121, 10.8%). In Wenzhou, eye injuries occurred mostly in summer (*n* = 136, 29.1%), and sharp object-related eye injuries accounted for the highest proportion (*n* = 98, 72.1%). In Changsha, eye injuries occurred mostly in winter (*n* = 272, 41.3%), and firecracker- and fireworks-associated eye injury accounted for the highest proportion (*n* = 73, 26.8%). The final VA was positively correlated with the initial VA (*r* = 0.641, *P* < 0.001) and the OTS (*r* = 0.582, *P* < 0.001).

**Conclusion:**

The age range of the susceptible pediatric population from rural areas was 3–8 years. Most eye injuries were penetrating, and the main cause of injury was a sharp object. Notably, the differences in the characteristics of eye injuries in the two areas were related to regional features.

## Background

Pediatric eye injuries account for the majority of uniocular visual disability and noncongenital unilateral blindness worldwide, especially in developing countries [1]. Each year, an estimated 3.3 to 5.7 million pediatric eye injuries occur worldwide, with a high incidence in rural children, of which 49% are serious enough to require inpatient hospitalization [2]. In recent years, the number of left-behind children in rural areas has increased markedly. A national survey in 2013 reported 61.025 million left-behind children [3], most of whom were raised by elderly caretakers. Low education levels, economic disadvantages and irresponsible child supervision all contributed to the alarmingly high incidence rate of eye injuries among rural children, with percentages as high as 46.7–48.1% [4, 5]. Visual outcomes for pediatric eye injuries have profound implications and significance, and treatment in children is more likely to be unsuccessful than treatment in adults [6].

However, it has been reported that 90% of eye injuries are preventable; therefore, identification of the frequency and spectrum of these injuries in a defined population and targeted educational and legislative efforts may be useful tools to minimize the incidence of eye injuries [7]. The epidemiology surrounding eye injuries has been reported in Beijing, Shanghai, Guangzhou, Chaoshan and Zhengzhou [8, 9]. However, there is not enough data to compare rates in the United States, Europe, and Australia, especially for pediatric populations. Moreover, a nationwide eye injury surveillance system has not yet been established in China, and there is an absence of representative clinic-based data in second- and third-tier Chinese cities. Wenzhou, located in southeast China, is an important regional center in southeastern Zhejiang Province; Changsha, located in south-central China, is the capital of Hunan Province. The resident population of Wenzhou has reached 9.25 million, and Changsha contains more than 7.9 million people. Both are second-tier, medium-sized cities, and their data are comparable to some extent (more detailed information is shown in Supplemental Table 1).

This study selected a tertiary hospital in each city (the Eye Hospital of Wenzhou Medical University and Xiangya Hospital of Central South University) to analyze the epidemiological characteristics of eye injuries among hospitalized rural children aged younger than 18 years over a 10-year study period and offer insight into injury prevention strategies for rural children.

## Methods

### Populations and procedures

This retrospective study collected data from hospitalized patients aged < 18 years who sustained serious eye injuries and were admitted to the Eye Hospital of Wenzhou Medical University or Xiangya Hospital of Central South University. Clinical records in the electronic medical record systems of the two hospitals were surveyed from January 2008 to December 2017. All cases with a principal diagnosis of ocular trauma were diagnosed according to the International Classification of Diseases, Tenth Revision, Clinical Modification (ICD-10-CM). Eye injury cases were searched using the ICD-10 code S05 in the electronic medical records.

Two researchers independently searched the records according to the above search strategy. Afterwards, the results of the two researchers were merged, and a third person re-check the inconsistent data. Finally, the three retrieval researchers excluded duplicated cases from the combined results. If the name, sex and date of birth were the same but the address was different, or if the name, sex and address were the same but the date of birth was different, the patients were considered the same person. Information including demographics, injury type, injury cause, time of presentation, initial visual acuity (VA) and final VA was recorded. The study followed the tenets of the Declaration of Helsinki. Because it was a retrospective study with de-identified information, informed consent was not required.

### Definitions

The Birmingham Eye Trauma Terminology (BETT) system was utilized to describe the type of mechanical injury and distinguish open-globe injuries (OGIs) from closed-globe injuries (CGIs) [10]. OGIs were further categorized as penetrating injury, perforating injury, intraocular foreign body injury or rupture. CGIs were further categorized as lamellar lacerations or contusions. The ocular trauma score (OTS) was used to evaluate the severity of eye injuries and the prognosis [11]. This index allowed the prediction of visual outcomes according to certain values (initial VA, rupture, endophthalmitis, perforating injury, retinal detachment, and relative afferent papillary defects). The method of OTS calculation is shown in Supplemental Table 2, and the scores relate to specific OTS categories. Based on the expected VA, the OTS was divided into five categories (1 = score of 0–44, 2 = score of 45–65, 3 = score of 66–80, 4 = score of 81–91, and 5 = score of 93–100) [11]. The initial and final VAs were categorized into five groups based on the OTS (1 = no light perception (NLP), 2 = light perception (LP)/hand movement (HM), 3 = 0.005–0.095, 4 = 0.1–0.4, 5 = ≥0.5). The final VA was obtained when the child was discharged from the hospital.

### Data and statistical analysis

Statistical analysis was performed using SPSS (18.0 version; IBM Corporation, Chicago, IL, USA). Normally distributed data were expressed as the means ± standard deviations (SDs). Children were divided into the age groups 0–2, 3–4, 5–6, 7–8, 9–10, 11–12, 13–14 and > 14 years old. Frequency analysis was performed using Pearson’s χ2 test. Correlation analysis (between the initial and final VA and the OTS and final VA) was performed using Spearman’s test. Two-tailed *P* values were calculated in all the analyses, and *P* < 0.05 was considered statistically significant.

## Results

This study examined 1125 children over the past decade; 467 children were admitted to the Eye Hospital of Wenzhou Medical University, and 658 children were admitted to the Xiangya Hospital of Central South University. Among them, 830 (73.8%) were males and 295 (26.2%) were females, yielding a male-to-female ratio of 2.81:1. The mean age of the children was 7.0 ± 4.1 years. The right eye was involved in 568 (50.5%) cases, and the left eye was involved in 534 (47.5%) cases. Binocular injury was observed in 23 children (2.0%).

### Distribution of eye injury characteristics

#### Age

**Fig. 1:**
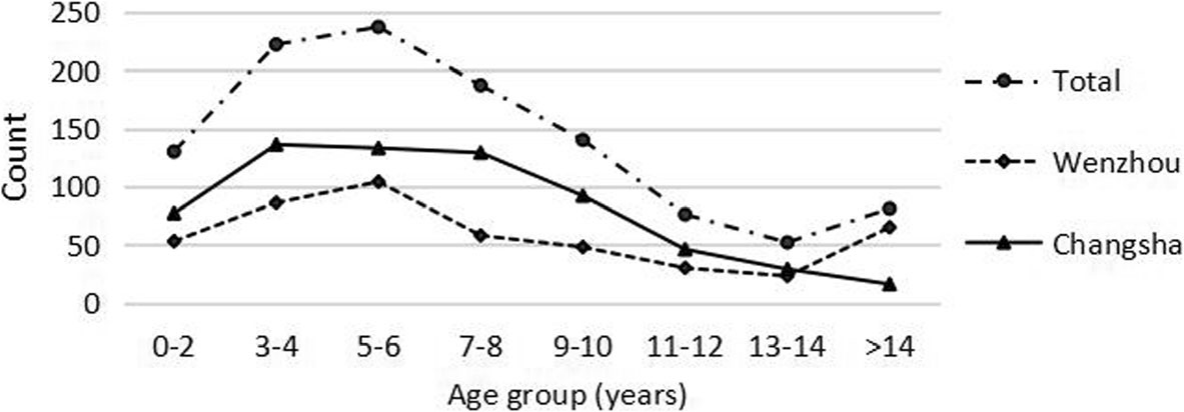
Distribution of eye injuries by age group and region

The age distribution showed that the peak age for the occurrence of eye injuries was 5 to 6 years (21.1%, *n* = 237), followed by 3 to 4 years (19.7%, *n* = 222) and 7 to 8 years (16.6%, *n* = 187) (Fig. 1). There was a significant difference in the age distributions between the two districts (χ^2^ = 63.121, *P* < 0.001). The proportion of children older than 14 years old was the lowest (*n* = 16, 2.4%) in Changsha but was relatively high (*n* = 65, 13.9%) in Wenzhou.

#### Injury types

According to the characteristics of the injuries, mechanical injuries had a higher frequency (*n* = 1007, 89.5%) than adnexal injuries (*n* = 91, 8.1%) and nonmechanical injuries (*n* = 27, 2.4%). Among the mechanical injuries, 783 children (69.6%) experienced OGIs, and 224 children (19.9%) experienced CGIs. The eye injuries in the two regions were mostly OGIs (Wenzhou = 409, 87.6%; Changsha = 374, 56.8%). Penetrating injuries (*n* = 689, 61.2%) accounted for the highest proportion in both regions, affecting 382 children (81.8%) in Wenzhou and 307 children (46.7%) in Changsha, followed by contusion injuries (*n* = 173, 15.4%) in 27 children (5.8%) in Wenzhou and 146 children (22.2%) in Changsha; adnexal injuries (*n* = 91, 8.1%) in 8 children (1.7%) in Wenzhou and 83 children (12.6%) in Changsha; and rupture injuries (*n* = 82, 7.3%) in 24 children (5.1%) in Wenzhou and 58 children (8.8%) in Changsha.

#### Injury causes

The top three injury causes were sharp objects (*n* = 544, 48.4%), blunt objects (*n* = 221, 19.6%) and fireworks (*n* = 121, 10.8%) (Table 1). In Wenzhou, summer was the main season for eye injuries (*n* = 136, 29.1%), with the highest proportion in July (*n* = 59, 12.6%). Sharp objects related to eye injuries accounted for the highest proportion (*n* = 98, 72.1%) in summer. In Changsha, winter was the main season for eye injuries (*n* = 272, 41.3%), with the highest proportion occurring in February (*n* = 131, 19.9%). Firecracker- and fireworks-associated eye injuries accounted for the highest proportion (*n* = 73, 26.8%) in winter.

**Table 1:** Cause of paediatric eye injuries by region

Cause of injuries	Wenzhou		Changsha		Total	
	n	%	n	%	n	%
Sharp object	323	69.2	221	33.6	544	48.4
Blunt object	69	14.8	152	23.1	221	19.6
Fireworks	14	3.0	107	16.3	121	10.8
Fall	15	3.2	42	6.4	57	5.1
Not recorded	11	2.4	45	6.8	56	4.9
Traffic accident	3	0.6	36	5.5	39	3.4
Other	7	1.5	15	2.3	22	2.0
Explosion	24	5.1	14	2.1	38	3.4
Animal	1	0.2	15	2.3	16	1.4
Burn	0	0.0	11	1.7	11	1.0
Total	467	100.0	658	100.0	1125	100.0

#### Time of presentation

**Fig. 2:**
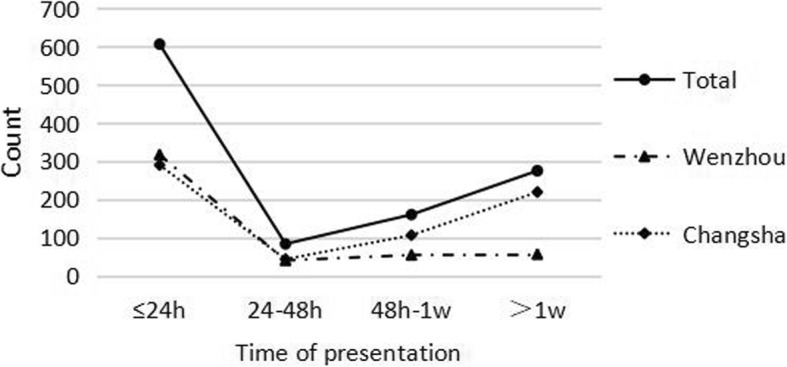
Distribution of time of presentation by region

In general, the majority of children with eye injuries (*n* = 607, 54.0%) presented to the hospital within 24 h, followed by admission after more a week after the injury (*n* = 275, 24.4%). There was a significant difference in the time of presentation between the two regions (χ^2^ = 84.842, *P* < 0.001). In Wenzhou, 67.9% (*n* = 317) of the children presented to the hospital within 24 h, and 12.0% (*n* = 56) presented to the hospital more than a week after the injury. In Changsha, 44.1% (*n* = 290) of the children presented to the hospital within 24 h, and 33.3% (*n* = 219) presented to the hospital over a week after the injury (Fig. 2).

### Distribution of the top three injury types

The top three mechanical eye injuries among the children were penetrating (*n* = 689, 68.4%), contusion (*n* = 173, 17.2%), and rupture injuries (*n* = 82, 8.1%). Between the two regions, the proportions of the three types of eye injuries were different (χ^2^ = 98.344, *P* < 0.001). The proportion of children with penetrating injury (*n* = 382, 88.2%) in Wenzhou was higher than that in Changsha (*n* = 307, 60.1%), but the proportions with contusion (27, 6.2%) and rupture injuries (*n* = 24, 5.6%) were lower than those in Changsha (contusion = 146, 28.6%; rupture = 58, 11.3%).

#### Age

**Fig. 3:**
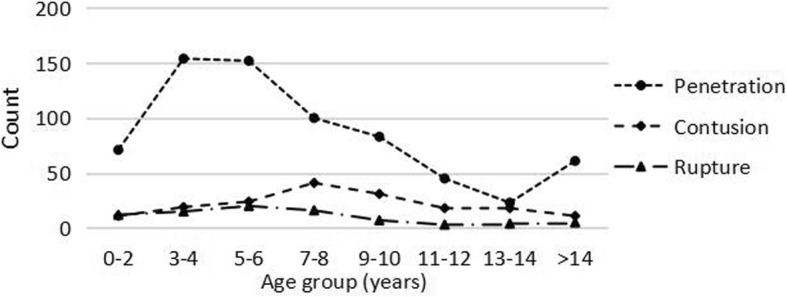
Distribution of the time of presentation for the top three mechanical injuries

In total, there were significant differences in the age distributions of these three types of injuries (χ^2^ = 51.134, *P* < 0.001). The age group with the highest incidence of penetrating injury was 3 to 6 years (*n* = 306, 44.5%). The number of penetrating injuries increased when children were older than 14 years old (*n* = 61, 8.9%). Contusion injuries were most likely to occur in children aged 7 to 10 years (*n* = 72, 41.6%). The majority of rupture injuries occurred in children aged 3 to 8 years (*n* = 51, 62.2%) (Fig. 3).

#### Injury causes

The highest percentage of penetrating injuries in children was caused by sharp objects (*n* = 389, 56.5%), and the most common injury object was a pair of scissors (*n* = 187, 48.1%), followed by an iron product (*n* = 55, 14.1%). The highest percentages of contusion injuries were caused by blunt objects (*n* = 76, 43.9%) and fireworks (*n* = 29, 16.8%), and the most common blunt object was a toy (*n* = 49, 28.3%). The highest percentages of rupture injuries were caused by sharp objects (*n* = 25, 30.5%) and fireworks (*n* = 17, 20.8%), and the most common sharp object was an iron product (*n* = 6, 24%).

#### Time of presentation

There were significant differences in the time of presentation between the three types of eye injuries (χ^2^ = 94.242, *P* < 0.001). Children with penetrating injury or rupture injury mostly presented to the hospital within 24 h (penetrating injury = 398, 57.8%; rupture injury = 66, 80.5%). Those with contusion injury mostly presented to the hospital more than a week after the injury (*n* = 78, 45.1%).

### Initial and final visual acuity associated with mechanical injury

**Fig. 4:**
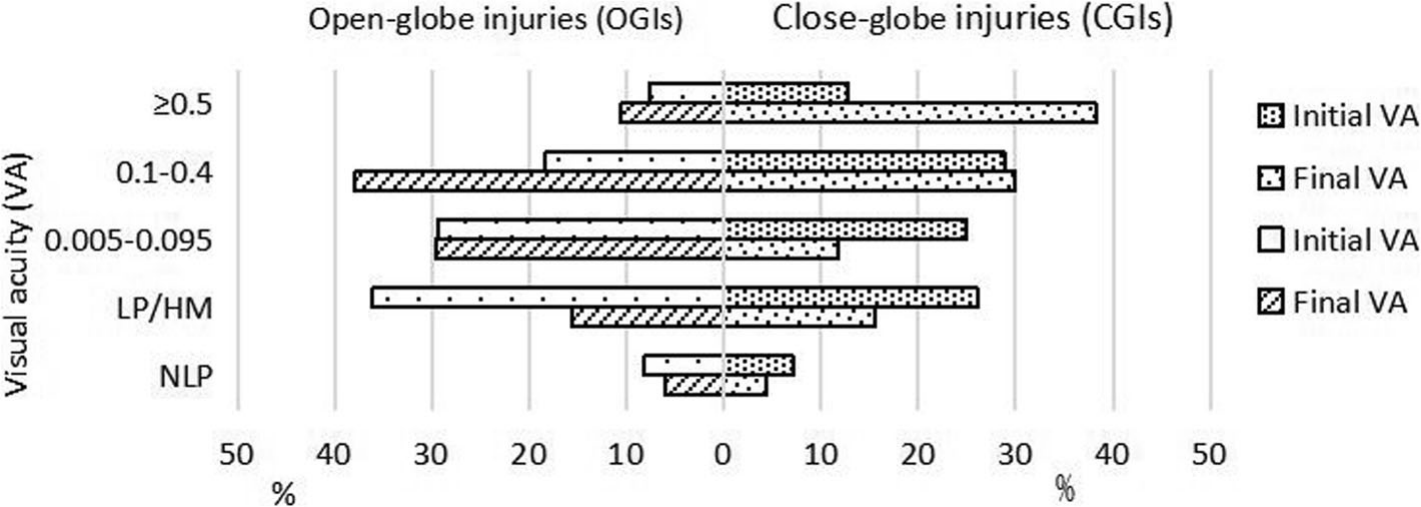
Relationship between the initial and final VA values in OGIs and CGIs (in %)

There were 268 children (3.46 ± 0.29 years) excluded from the VA evaluation. Of the 739 children included in the analysis, 559 (75.6%) children experienced OGIs, and 180 (24.4%) experienced CGIs. The statistical analyses revealed significant differences in the distributions of initial and final VA values between children with OGIs and CGIs (χ^2^ = 16.261, *P* = 0.003; χ^2^ = 81.171, *P* < 0.001). In children with OGI, an initial VA ≥0.5 was present in 43 (7.7%) children, an initial VA < 6/60 was present in 413 (73.9%) children, a final VA ≥0.5 was present in 59 (10.6%) children, and a final VA < 6/60 was present in 288 (51.5%) children. In children with CGI, an initial VA ≥0.5 was present in 23 (12.8%) children, an initial VA < 6/60 was present in 105 (58.3%) children, a final VA ≥0.5 was present in 69 (38.3%) children, and a final VA < 6/60 was present in 57 (31.7%) children (Fig. 4).

Overall, 43.2% (*n* = 319) of the children showed improved VA, 47.6% (*n* = 352) maintained the same VA, and 9.2% (*n* = 68) showed deteriorated VA. The final VA in children was significantly better than the initial VA (OGI: χ^2^ = 378.672, *P* < 0.001; CGI: χ^2^ = 132.327, *P* < 0.001). There was no significant difference in the VA changes between the OGI and CGI groups (χ^2^ = 3.975, *P* = 0.137). Among the children with OGI, the final VA was improved in 41.3% (*n* = 231), remained the same in 48.7% (*n* = 272), and deteriorated in 10.0% (*n* = 56). Of the 56 children with VA deterioration, 30 (53.6%) presented to the hospital within 24 h. Among the children with CGI, the final VA was improved in 48.90% (*n* = 88), remained the same in 44.40% (*n* = 80), and deteriorated in 6.70% (*n* = 12). Of the 12 children with VA deterioration, 7 (58.3%) presented to the hospital more than a week after the injury.

### OTS and visual prognosis

A comparison of the final VA and initial VA is presented in Table 2. The initial VA was correlated with the final VA (*r* = 0.641, *P* < 0.001). The OTS was calculated for 739 children with mechanical injuries, and the OTS and the final VA were also positively correlated (*r* = 0.582, *P* < 0.001) (Table 3).

**Table 2:** Final VA compared with the initial VA

VA	Initial VA		Final VA	
	Frequency	(%)	Frequency (%)	(%)
NLP (%)	59	8.0	42	5.7
LP/HM (%)	249	33.7	116	15.7
0.005–0.095 (%)	210	28.4	187	25.3
0.1–0.4 (%)	155	21.0	266	36.0
≥0.5 (%)	66	8.9	128	17.3

*VA* visual acuity, *NLP* no light perception, *LP* light perception, *HM* hand movement

**Table 3:** Correlation of the final VA with the OTS in the OTS study group (739 eyes)

OTS		Final VA category					
Raw score	Category	NLP(%)	LP/HM(%)	0.005–0.095(%)	0.1–0.4(%)	≥0.5(%)	Total(%)
0–44	1	33 (4.47)	39 (5.28)	52 (7.03)	20 (2.71)	2 (0.27)	146 (19.76)
45–65	2	9 (1.22)	66 (8.94)	93 (12.58)	93 (12.58)	28 (3.79)	289 (39.11)
66–80	3	0 (0)	10 (1.35)	39 (5.28)	128 (17.32)	51 (6.90)	228 (30.85)
81–91	4	0 (0)	1 (0.14)	3 (0.41)	23 (3.11)	27 (3.65)	54 (7.31)
92–100	5	0 (0)	0 (0)	0 (0)	2 (0.27)	20 (2.70)	22 (2.97)
Total		42 (5.69)	116 (15.71)	187 (25.31)	266 (35.99)	128 (17.32)	739 (100)

*VA* visual acuity, *NLP* no light perception, *LP* light perception, *HM* hand movement

## Discussion

Internationally, 20 to 59% of all eye injuries occur in children [6]. Pediatric eye injuries are characterized by high morbidity and high blinding rates and are mostly treatable and preventable. In our study, we found that 69.6% of the children required hospitalization for OGI, similar to results reported by others in China [7, 12] and India [2, 13]. In contrast with our results, a study conducted in Lithuania by Puodžiuvienė et al. [14] found that CGI was the most common type of eye injury in children. Similar results were also presented in other studies [15–17].

Our study also found that children aged 5 to 6 years had the highest proportion of pediatric eye injuries (21.1%), followed by children aged 3 to 4 years (19.7%) and children aged 7 to 8 years (16.6%). Yardely et al. [17] reported that children in the up to 5 year age group were the most susceptible to injury. Additionally, Bunting et al. [18] and Wang et al. [12] reported similar results. However, Edita et al. [14] found that 72.8% of injuries occurred in children older than 7 years, and Bućan et al. [15] reported that the highest incidence of eye injuries occurred among children aged 10 to 14 years. These different results could be explained by the types of pediatric eye injuries in different regions. In our study, penetrating OGIs occurred most frequently in early school-aged children (3 to 6 years), and contusion CGIs occurred most frequently in school-aged children (7 to 10 years), which was consistent with the age characteristics reported by the above scholars.

In addition, we found that there was an increase in the number of eye injuries in children older than 14 years, and a large proportion of children were injured by iron products in Wenzhou. These results may be related to a high number of industrial factories and many small, family-style factory workshops in Wenzhou. Several studies have found that work-related injuries are the most common type of eye injury, and metallic objects remain the leading cause of object-related eye injury [19–21]. Zhang et al. [19] found that metallic objects were the main cause of eye injury in children aged > 14 years. In our study, children in this age group were about to enter adulthood; in addition, they came from rural areas. Therefore, we speculate that children in this age group may enter the workforce to earn money. A low education level, lack of safety awareness and lack of protective eyewear may lead to the occurrence of eye injury. Additionally, we found that male children were more likely to be injured than female children, consistent with data from other studies [16, 22, 23].

In Changsha, the majority of injuries occurred during winter, and most of the injury causes were firecracker- and fireworks-related. Eye injuries occurred most frequently in February, consistent with other studies [12, 24]. Several factors may account for this phenomenon. First, the Spring Festival usually occurs at the end of January. Second, more than 80% of all fireworks and firecrackers are produced in Liuyang city, a subsidiary of Changsha city, Hunan Province, which increases exposure to fireworks and firecrackers [12]. Additionally, the legislation on fireworks and firecrackers was issued in January 2018 and was applied to rural areas after March 2018, which was later than the time period of this study [25]. Third, most of the children from the rural districts do not know how to protect themselves properly when lighting fireworks. Eye injuries in Wenzhou occurred least frequently in winter and most frequently in summer, especially in July, and most of the injuries were caused by a sharp object. The government imposed restrictions on fireworks and firecrackers through legislation in August 2006 in Wenzhou, which may have led to a reduction in this kind of injury [26]. Bućan et al. [15] and Podbielski et al. [27] both found that the summer vocation months accounted for the greatest percentage of eye injuries throughout the year, similar to our study. During the summer period, children have the opportunity to spend more time playing and are thus more readily exposed to potentially dangerous objects, especially scissors, than during the school year. Furthermore, parents are busy and have difficulty in caring for their children during holidays.

In another study, we found that 607 (54%) children with eye injuries presented to the hospital within 24 h, and 275 (24.4%) children presented to the hospital more than a week after the injury. Yehia et al. [28] reported that 56% of children presented to the hospital within 24 h of their eye injury, and 6.6% of children presented to the hospital after more than a week. Aghadoost et al. [29] found that 69% of children presented to the hospital within 24 h. There was a significant difference in the time of presentation between Wenzhou and Changsha. The main reason could be that children in Wenzhou suffered from penetrating injuries (81.8%) more often than those in Changsha (61.2%), and children in Changsha suffered from contusion injuries (22.2%) more often than those in Wenzhou (5.8%). Most of the children who had penetrating (57.8%) or rupture injuries (80.5%) presented to the hospital within 24 h, while children who had contusion injuries (45.1%) mostly presented to the hospital more than a week after the injury.

Of the 739 children with mechanical injuries, we found that 73.9% of the children with OGI had a VA < 6/60 at presentation, and 51.5% of the children had a final VA < 6/60. Minder et al. [16] found that in children with OGI, an initial VA < 6/60 was reported in 68% and a final VA < 6/60 was reported in 59%. Compared with the initial VA, improvement in the VA was found in 319 (43.2%) children, and VA remained the same in 352 (47.6%) children, which differs from Bućan’s study [15]. Additionally, there was no significant difference in the distribution of VA changes between the OGI and CGI groups. In our study, children with CGI with poor vision mostly presented to this hospital more than a week after the injury. We speculated that the time of presentation for CGI would be later than that for OGI, which may delay optimal treatment. Narang et al. [30] reported that the final VA was significantly worsened in eyes in which the primary repair was delayed beyond 24 h.

The OTS serves as a prognostic tool to predict visual outcomes in patients after eye injuries. Some studies have verified the reliability and predictability of this assessment system [12]. Several studies have reported that the OTS can be utilized to accurately predict outcomes [22, 31, 32]. However, Oiticica et al. [33] found that the OTS had limited value in predicting long-term VA in children. Good initial vision (*r* = 0.641, *P* < 0.001) and a high OTS (*r* = 0.582, *P* < 0.001) were statistically correlated with good final VA in our study, but the correlation coefficient was not very high, consistent with Cao’s study [7]. This result may be related to the poor coordination of some children in the examination of initial vision and relative afferent pupillary disorders, which may affect the accuracy of the judgment.

The study had potential limitations related to the retrospective study design. First, the VA at the follow-up was not recorded. Hence, we were unable to evaluate the stability of the VA. Second, due to a lack of coordination in some pediatric patients, VA data were not recorded in some children. Third, the data are not a complete representation of all eye injuries suffered by the pediatric population in rural Wenzhou and Changsha. However, most of the children with pediatric injuries would have been hospitalized in the hospitals we selected in each location. Finally, this study population included only rural children requiring hospitalization, which limits generalization to other settings.

## Conclusions

In summary, most of the eye injuries in hospitalized children from rural areas were penetrating injuries and occurred between the ages of 3 and 8 years. The main cause of injury was a sharp object, especially scissors. Parents should be reminded to be aware of sharp object-related injuries and strengthen their supervision. Notably, the differences in the characteristics of eye injuries in the two areas were related to regional features. In Changsha, a combination of efforts to raise public awareness and the implementation of appropriate legislation should be performed to prevent fireworks- and firecracker-related eye injuries. A previous systematic review showed that the trauma incidence rate decreased by 87% after enforcing restrictive fireworks laws. The main factor was the legal restrictions on the personal use of fireworks [34]. In Wenzhou, due to the large number of factories, staff are likely to be injured by iron products. Therefore, the relevant departments should focus on the safety of the production environment, popularize safety knowledge, guide the staff to correctly utilize protective safety equipment, and reduce the occurrence of such eye injuries in children. Several studies have shown that the provision of appropriate protective eyewear reduces the incidence of eye injuries in the workplace [35, 36]. The majority of pediatric eye injuries are preventable, which reflects the importance of targeted prevention measures.

## Supplementary information

**Additional file 1: Supplemental Table 1.** Objective characteristics for Wenzhou and Changsha in 2018. **Supplementary Table 2.** Computational method for deriving the OTS score.

## Data Availability

The datasets analysed in this study are available from the corresponding author (Yanyan Chen, cyy@mail.eye.ac.cn; Minhui Dai, 810835852@qq.com) upon reasonable request.

## Abbreviations

CGI: Closed-globe injuries
OGI: Open-globe injuries
OTS: Ocular trauma score
VA: visual acuity
