# Supplementary material for: Epidemiology of pediatric eye injuries requiring hospitalization in rural areas of Wenzhou and Changsha, China: a 10-year retrospective study
Source: BMC Ophthalmol. 2020 Mar 14;20:100. doi: 10.1186/s12886-020-01363-7 (PMC7071590; doi:10.1186/s12886-020-01363-7)
Supplement: Supplementary file 1 — Additional file 1: Supplemental Table 1. Objective characteristics for Wenzhou and Changsha in 2018. Supplementary Table 2. Computational method for deriving the OTS score. [file 12886_2020_1363_MOESM1_ESM.pdf]

Supplemental Table 1. Objective characteristics for Wenzhou and Changsha in 2018

City	Geographical position	Climate	Land area (square kilometers)	Resident population (million)	Rural population (million)	GDP (billion)	GDP per capita	Per capita disposable income	Per capita disposable income of rural residents	Proportion of manufacturing industry
Wenzhou	Southeastern Zhejiang Province	Subtropical monsoon; Annual average temperature is 18.9℃	11613	9.25	2.78	￥6006.2	￥65055	￥46920	￥27478	35.80%
Changsha	Northeastern Hunan Province	Subtropical monsoon; Annual average temperature is 18.4℃	11819	7.90	2.23	￥1021.01	￥131207	￥44647	￥27360	18.20%

Supplementary table 2. Computational method for deriving the OTS score

Initial visual factor		
A. Initial raw score (based on initial visual acuity)	NPL =	60
	PL or HM =	70
	0.005 to 0.095 =	80
	0.1 to 0.4 =	90
	≥0.5 =	100
B. Globe rupture		-23
C. Endophthalmitis		-17
D. Perforating injury		-14
E. Retinal detachment		-11
F. Relative afferent papillary defects (RAPD)		-10
Raw score sum = sum of raw points		
